# Exploring the length scales, timescales and chemistry of challenging materials (Part 1)

**DOI:** 10.1098/rsta.2022.0353

**Published:** 2023-10-16

**Authors:** Martin C. Wilding, Andrea Sella, Christopher A. Howard, Ana Jorge Sobrido, C. Richard A. Catlow

**Affiliations:** ^1^ Cardiff Catalysis Institute, School of Chemistry, Cardiff University, Cardiff CF10 3AT, UK; ^2^ Department of Physics and Astronomy, University College London, London WC1E 6BT, UK; ^3^ Department of Chemistry, University College London, London WC1E 6BT, UK; ^4^ School of Engineering and Materials Science, Queen Mary University of London, London E1 4NS, UK

**Keywords:** glasses, polyamorphism, neutron diffraction, X-ray diffraction, extreme conditions, liquid structure

## Abstract

This themed issue explores the different length scales and timescales that determine the physics and chemistry of a variety of key materials, explored from the perspective of a wide range of disciplines, including physics, chemistry, materials science, Earth science and biochemistry. The topics discussed include catalysis, chemistry under extreme conditions, energy materials, amorphous and liquid structure, hybrid organic materials and biological materials. The issue is in two parts, with the present part exploring glassy and amorphous systems and materials at high pressure.

This article is part of the theme issue 'Exploring the length scales, timescales and chemistry of challenging materials (Part 1)'.

## 1. Introduction

The origin of this issue was a meeting held in June 2022 that honoured the scientific contributions of our friend and colleague Prof. Paul F. McMillan, who sadly passed away earlier that same year and who was a major contributor to solid-state science, materials chemistry, geochemistry and high-pressure physics. The meeting presented new, emerging science in fields that McMillan had influenced.

The physical and chemical properties of materials undoubtedly reflect interactions over different length scales and timescales, with new understandings often gained via advanced experimental techniques that can include neutron and X-ray scattering as well as the vibrational spectroscopy approaches that were widely employed by McMillan. Invariably, a deeper understanding of the influence of the atomistic and molecular structural influences requires computational modelling, and combinations of experimental and theoretical techniques are presented in these two volumes and underscore the value of such combined approaches and connections between different scientific disciplines.

The field of amorphous and glassy materials is particularly fascinating, especially the influence of local structures on the structural relaxation of glass-forming liquids. Importantly, the dynamics of glass-forming liquids reflect the influences of the variable distances and timescales that are explored in this issue. The concept of *polyamorphism* is particularly interesting, as in the study of Y_2_O_3_-Al_2_O_3_ glasses close to the YAG composition [1]. This early work was controversial, but several subsequent studies have established the thermodynamic and structural origin of polyamorphism in this aluminate system using the same combined experimental and theoretical approaches that are outlined in this issue [2–4].

Polyamorphism (glass polymorphism), in the strictest sense, is the formation of different structured glasses by different synthesis routes and as such may seem intuitive and unremarkable. A classic example, and indeed the typical example, is of glasses formed at room pressure and glasses formed by pressure-induced amorphization [5,6], which differ in density and local structure. However, in some cases there are abrupt changes between these different glass structures, suggesting a first-order phase transition between different structured liquids and the presence of critical-like phenomena in the metastable, supercooled liquid regime. The first report of the presence of such a liquid–liquid phase transition (LLPT) was in a study of amorphous ice, with a spectacular transition between high-density amorphous (HDA) ice produced by pressure-induced amorphization and a lower-density form, LDA [7]. In the Y_2_O_3_-Al_2_O_3_ system, the underlying LLPT is reflected in the production of two glasses of different densities produced when a high-temperature liquid is rapidly quenched. It is important to emphasize that the two glasses are identical in composition and are not, therefore, a representation of liquid immiscibility, but rather of differences in the entropy and density of high- and low-density configurations that can be described by critical-like phenomena with the LLPT terminating in a critical point which is often predicted to occur at experimentally inaccessible pressures and temperatures. Although hard to observe directly, the differences in density and entropy responsible for LLPTs can be reflected in thermodynamic anomalies, including the familiar density maximum at 4°C in water, which can be observed directly and which suggest that polyamorphism and the associated LLPTs are present in a variety of different systems [8].

In the contribution from Domagoj Fijan & Mark Wilson [9], the appearance and evolution of the thermodynamic anomalies that are associated with polyamorphism are explored. Computer simulations are used to explore several candidate polyamorphic systems, achieved by systematically varying the key modelling parameters. Although the density anomaly in water is the most famous, similar maxima are reported in other systems, including Si and Ge and also SiO_2_ and GeO_2_, Different modelling approaches are applied to these systems, which can be categorized roughly as ionic or covalent, and the emergence of thermodynamic anomalies in, for example, compressibility and heat capacity can be observed by systematically varying parameters such as ion polarizability across phase space and the critical-like phenomena characteristic of polyamorphic phase transitions identified.

The key structural and thermodynamic properties associated with polyamorphism often occur in metastable regions of phase diagrams, in deeply supercooled regimes and often at high pressure. This means that these characteristic features are experimentally inaccessible and has added to the controversy associated with polyamorphism and LLPTs. While a lot of information can be obtained from recovered glasses, glasses result from a complex trajectory through the supercooled regime involving partial structural relaxation until kinetic arrest. Therefore, glassy structures are not necessarily representative of either stable or supercooled liquids. To explore liquid structures, sample environments have been developed that allow direct measurement of liquid structures using diffraction methods. The total scattering for liquids is weak, and for liquids contained in conventional furnaces the crystalline contributions from these sample environments can dominate the diffraction patterns. A novel alternative to bulky furnaces is the containerless approach in which these sample environments are eliminated altogether and liquid drops accessed directly. A commonly used containerless technique, which is discussed in two studies in this volume, is the aerodynamic levitation furnace [10,11], illustrated on the cover of this themed issue. These levitation furnaces comprise a bespoke conical nozzle in which the sample is suspended by a gas jet. The sample bead, typically 2–3 mm in diameter, is laser heated until molten. These furnaces can be readily integrated into beamlines at large-scale facilities and diffraction data from either neutron or high-energy X-ray sources can be obtained. As well as the obvious advantage of minimal background contribution, the liquids are not in contact with the nozzle and so heterogeneous nucleation is avoided and the liquids can be supercooled and even quenched to glasses. Furthermore, the composition of the levitation gas can be controlled by gas-mixing and systems with mixed valance elements can be studied.

In the contribution of Shi *et al.* to this issue [12], high-energy X-ray diffraction (XRD) measurements on levitated liquid collected at the Advanced Photon Source (APS) of the Argonne National Laboratory are presented. Here the aerodynamic levitation furnace forms part of the infrastructure at beamline 6-ID-D, and data have been collected for liquids in the CaO-FeO-Fe2O3 system under different partial pressures of oxygen. Iron is one of the most important mixed-valent elements and the different oxidation states strongly influence the physical properties of iron-bearing liquids, not least geologically important magmatic and volcanic liquids. The diffraction data obtained by Shi *et al.* are combined with EPSR modelling and the partial structural contributions of the ferrous components evaluated. It is shown, for instance, that there is a systematic increase in Fe^2+^-O coordination number as the ferrous content is increased, while the Fe^3+^-O coordination number remains constant regardless of the ferric : ferrous ratio. This suggests that the structure of this calcium ferrite liquid is influenced by the distorted coordination environment around Fe^2+^.

In the study of Drewitt *et al.* [13], which also uses an aerodynamic levitation furnace, the structure of iron-bearing aluminate liquids is reported. In this contribution, the containerless sample environment is integrated into the D4c diffractometer at the Institut Laue-Langevin in Grenoble. Since neutrons are used, isotopic substitution can be used to unequivocally determine the iron contributions to the total scattering. This study also employs classical and *ab initio* molecular dynamics to model the liquid structures. In this case the iron is all divalent, yet it is still shown to occupy a variety of Fe-O coordination environments and clearly illustrates that the single, rigid octahedral and tetrahedral motifs that are so often used to model iron-bearing liquid structures are not necessarily appropriate.

Glasses and amorphous materials are formed in a variety of systems which extend beyond the compositions of traditional silicate systems and accordingly have many different applications. One important class of glasses are the bioactive glasses [14] which contain calcium, fluorine and phosphate ions that can be readily incorporated into the human body; this means that they can be used clinically in, for example, bone repair. Because bio-glasses are glassy, they are not restricted in composition and can be modified and the biocompatibility optimized. In the paper by Jamie Christie [15], results from the computer modelling of several bioactive glasses are presented. The influence of network connectivity and medium-range order is evaluated, and biocompatibility is correlated with the formation of clusters of phosphate coordination polyhedra. The formation of regions of fluoride clusters, chemically bonded to sodium and calcium ions, is also correlated with increased bioactivity.

Amorphous materials are encountered in many different classes of materials; amorphous materials include materials that are considered glassy, but the distinction between materials that are considered amorphous and those that are glasses is not well defined. Glasses can be shown to have a calorimetric glass transition observed when they are heated, and this is assumed to be a kinetic feature representing the temperature dependence of the timescale of structural relaxation. The compositions of such glasses extend beyond traditional silicate systems, and two contributions to this special issue explore glassy behaviour in carbonate systems. Carbonate glasses are rare, and amorphous carbonates which have high water content are more commonly encountered. Amorphous calcium carbonates (ACC) are produced by biogenic processes and are precursors to more stable crystalline carbonates; because of their high water content and low temperature, it has been generally assumed that they are not glasses and that when heated they dehydrate and crystallize. In the study by Hess *et al.* in this volume [16], a calorimetric study of an amorphous calcium magnesium carbonate (ACMC) demonstrates the presence of a glass transition in this amorphous material. Hess *et al.* use flash calorimetry, a technique that enables much faster scanning rates to be used (up to 3000 K s^−1^) than in differential scanning calorimetry, to identify the glass transition that occurs before diffusion results in crystallization. This short-timescale probe indicates that ACMC is a structural glass with relaxational properties indistinguishable from those of the rare potassium magnesium carbonate glass also studied by this group.

The study of Weindorfer *et al.* [17] is a calorimetric study of the K-Mg carbonate glass first reported by Eitel and Skaliks in 1929 [18]. Since this rare glass is only produced at elevated pressure, this contribution offers a nice segue between the two sections of this themed issue. This carbonate glass was generally considered to be an anomaly and received little attention until it became apparent that carbonate materials can be encountered in the deep Earth. High pressure is required to ensure that volatile species remain dissolved at high temperatures; water is readily dissolved in the K-Mg carbonate glass, and indeed it is difficult to produce completed anhydrous carbonate glasses. Weindorfer *et al*. have collected glass transition temperature data for a range of K-Mg carbonate glasses synthesized at high pressure and with a range of water contents. The glass transition temperature shifts progressively to lower temperatures as the water content is increased, such that for the most water-rich glass the glass transition is at 85°C, significantly below that identified for the ACMC and further blurring the distinction between materials considered glassy and those considered amorphous. This study indicates that these water-rich, carbonate liquids can be active transport vectors in many different geological settings, ranging from the deep mantle to sub-surface crustal environments.

Materials at high pressure were one of McMillan's many research interests [19–21]. High-pressure research encompasses a range of disciplines. The complex physics and chemistry that influence the behaviours of high-pressure materials, geochemical and geophysical processes and biological processes, including the survival of bacteria at high pressure [22,23], all reflect interactions on different length scales and timescales, discussed in this themed issue and requiring the application of both neutron and X-ray scattering techniques, specialized high-pressure sample environments (diamond anvil cells and multi-anvil apparatus) and computational modelling.

The behaviour of perovskite structured materials at high pressure is a particularly rich area of extreme-conditions research. In the *in situ* high-pressure neutron and XRD study of Craig Bull and colleagues, the high-pressure behaviour of PrCrO_3_ is explored [24]. The structure of this important magnetoelectric material is evaluated at room pressure over a range of temperatures; in this case the structure is relatively insensitive to changes in temperature, as evidenced by the limited tilt of the constituent polyhedra. The response to pressure of up to 5.6 GPa is more interesting: although there is no change in the crystal symmetry, there are changes in unit cell parameters and the compression is anisotropic, the crystallographic a-axis being much more compressible. It is possible that this material softening indicates the onset of a phase transition. High-pressure Raman spectroscopy also shows softening of specific modes and discontinuous behaviour that also suggests the onset of a secondary phase transition at higher pressures.

The formation of different structures at different pressures and temperatures with first-order transitions defining the boundaries between these phases, *polymorphism*, is one of the characteristic responses of crystalline materials to high pressure. Binary metal oxides have a large number of different polymorphs, and pressure-induced changes in oxygen positions can have a dramatic effect on electrical conductivity. Two synchrotron X-ray techniques, combined with DFT modelling, are used by Sneed *et al.* [25] in their high-pressure study of SnO_2_ using a CO_2_ laser-heated diamond anvil cell. Diffraction and X-ray absorption spectroscopy data were collected at the APS and show that the ambient-pressure rutile-structured SnO_2_ undergoes a series of phase transitions, first adopting a CaCls structure and then at the highest pressure a PbF_2_ cubic structure. An a-PbO structure, common in oxides, is metastable relative to these other phases, although it becomes more stable and energetically favours high temperature and high pressure, illustrating incredibly complex phase behaviour for a seemingly simple material.

Nitride materials have numerous possible technical applications, although their synthesis is challenging. High-pressure, high-temperature techniques are used to synthesize these materials, and consequently the chemistry of nitride systems at high pressure has been studied extensively [26,27]. In the contribution of Yuan *et al.* [28] the synthesis of La-Na-N materials at high pressure is described, where sodium azide (NaN_3_) is used as the source of nitrogen. For a range of LaN : NaN_3_ compositions, two different structure types have been produced at pressures of 8 GPa and temperatures of 800°C in a multi-anvil press. Tetragonal La_1−*x*_N_3*x*_N phases with distorted, rock salt-related structures are produced with *x* between 0.1 and 0.14. These have an excess of cations over anions, accommodated by excess cations in interstitial sites or the formation of vacancies. For higher values of sodium, the behaviour is apparently two-phase with two cubic phases, the distorted rock salt structure having increased distortion at higher Na content and forming a cubic phase which coexists with another cubic rock salt structure, although with a smaller cell parameter. This study, which is the first report of the synthesis of ternary phases in the La-Na-N system, suggests a high-pressure phase diagram rich in these defect rock salt structures.

Spinel structures are also commonly encountered at high pressure, and Andreas Zerr presents a study of a tin nitride, synthesized *in situ* in a laser-heated diamond anvil cell [29]. The starting materials are elemental tin and molecular nitrogen, and the application of both high pressure and high temperature are shown to result in formation of the γ-Sn_3_N_4_ spinel at 10–20 GPa and 2000°C. The structure of the recovered material is confirmed by XRD. Raman spectroscopy is used to determine the Grüneisen parameters of this spinel and the thermal shock resistance of this technically important material.

The elastic moduli and refractive index of a spinel-structured germanium nitride are presented in the contribution of Chen Hui Li *et al.* [30] This material had been synthesized independently by two groups, the group including McMillan [31,32] at Arizona State University and the group at Mainz [33]. In the study presented here by Li *et al.,* both the bulk and the shear moduli are determined by laser ultrasonics and Brillouin light spectroscopy. These experiments are combined with first-principles DFT calculation. Using γ-GaN synthesized in a multi-anvil press, polished samples had been prepared for measurement of longitudinal and Rayleigh wave velocity, and although the DFT calculation underestimates the experimentally determined value of the bulk modulus, the calculated shear modulus matches the values determined from laser ultrasonics and Brillouin light spectroscopy. Importantly, this contribution demonstrates that the moduli can be determined for polycrystalline materials, if porosity can be taken into account. The combined data have been used to determine the refractive index of γ-GaN, which also matches the value from DFT calculations.

Many materials show exceptional physical properties at high pressure and one particularly interesting material is boron carbide, a super-hard, lightweight material that has a large number of potential technical applications. However, this intriguing material shows a number of anomalous properties, including a loss of shear strength at high pressure, that may limit its application. The study presented by Somayazulu *et al.* [34] provides information on the elastic properties of boron carbide. Their study provides the equation of state determined by XRD measurements at HPCAT of the APS, made *in situ* using a laser-heated diamond anvil call, expanding the pressure and temperature range of the equation of state to 50 GPa and 2500 K.

The contributions to this first part of the themed issue give merely a snapshot of the current research in two areas. They illustrate the importance of combining experimental techniques and theoretical calculations and underscore how different length scales and timescales influence the properties of complex materials. We would like to thank all of the contributing authors for giving their time and energy to enable the publication of this exciting collection of articles. We are also grateful to the Royal Society Editorial staff for their assistance in producing this volume.

This theme issue was put together by the Guest Editor team under supervision from the journal's Editorial staff, following the Royal Society's ethical codes and best-practice guidelines. The Guest Editor team invited contributions and handled the review process. Individual Guest Editors were not involved in assessing papers where they had a personal, professional or financial conflict of interest with the authors or the research described. Independent reviewers assessed all papers. Invitation to contribute did not guarantee inclusion.

## Data Availability

This article has no additional data.
